# The combined analysis of hair samples with fly larvae for the forensic identification of skeletonized human remains

**DOI:** 10.1007/s11419-026-00758-3

**Published:** 2026-01-27

**Authors:** F. Palazzoli, P. Verri, D. Vandelli, A.L. Fabris, V. Castagnetti, G. Gualandri, A.L. Santunione

**Affiliations:** 1 Institute of Legal Medicine, Department of Biomedical, Metabolic and Neural Sciences, University of Modena and Reggio Emilia, Modena, Italy; 2 Legal Medicine and Bioethics, Azienda USL-IRCCS di Reggio Emilia, Reggio Emilia, Italy

**Keywords:** Larvae, Hair, Phenobarbital, Phenytoin, Forensic toxicology, Entomotoxicology

## Abstract

**Purpose:**

In the present work, we report the combined use of hair and fly larvae samples as a valuable approach for obtaining additional toxicological evidence in cases of skeletonized corpses. In highly decomposed human remains, the absence of conventional biological specimens (e.g. blood, urine, and organs) requires alternative matrices to base the forensic investigation upon. However, it is challenging to find the most suitable medico-legal approach in these cases.

**Methods:**

Hair and fly larvae samples were collected from two independent cases and processed by solid phase extraction using a similar procedure. Analyses were performed using liquid chromatography tandem mass spectrometry.

**Results:**

Two anticonvulsants were found in the samples but in a different pattern: phenobarbital in case 1, and phenobarbital in association with phenytoin in case 2.

**Conclusions:**

The results of this toxicological approach combining two different samples, hair and larvae, in addition to circumstantial and autopsy findings, proved to be paramount in the medico-legal assessment of the cases herein described, leading to the successful identification of the subjects by DNA analysis. We thus hope that the approach and findings reported in this work contribute to the growing entomotoxicology science and its applicability to real complex forensic cases, especially considering that, to the best of our knowledge, this is the first article describing the determination of phenytoin in insects for this purpose.

## Introduction

In forensic investigations, conventional evidence-like specimens, such as body fluids, soft tissues, and organs, are used to understand the cause of death. These matrices provide information about a person’s demise, which is critical for legal investigations. However, such specimens are absent in bodies at advanced stages of decay, thus complicating the execution of toxicological analyses and medico-legal interpretation. In these cases, alternative matrices, such as hair, have been shown to be valuable assets [1, 2].

Hair fibers are more stable than conventional body tissues, allowing a wide time window for analysis. Considering drugs are passively incorporated into growing hair fibers, a historical record of past use or exposure to substances can be achieved. However, this matrix is unsuitable for estimating recent drug intake or exposure for forensic purposes due to the hair growth rate (1 cm/month on average) and is thus limited to exposures that took place at least 1 month prior to sample collection [3, 4]. In that regard, an additional type of sample that can provide valuable toxicological information from a more recent timeframe is entomotoxicological matrices, i.e. insects at different developmental stages. Necrophagous insects feed on cadaveric tissues and drugs enter their system as a consequence; such insects can then be collected and serve as a source of information about the substances to which the person was recently exposed [5]. This approach, nowadays known as forensic entomotoxicology, was introduced in the 1970s as a branch of forensic entomology, only gaining momentum in recent years, although it remains underexplored [6]. The use of such insects is particularly interesting in highly decomposed human remains because they are usually still available whereas other bodily fluids and tissues cannot be sampled anymore.

To date, several studies describing the use of hair fibers or insects for the detection of xenobiotics in highly decomposed corpses have been reported in the literature [7–10], albeit only few of those combined these two sample types [1]. Indeed, the correlation between the concentration of drugs found in insects and those present in cadaveric organs is still unclear and has not been extensively studied [2]. As a result, more studies are warranted to shed light on the combined use of such specimens in forensic investigations involving human remains in advanced stages of decomposition. It is important to mention that entomotoxicological matrices are generally unsuitable to infer the cause of death but can definitely serve as an additional source of toxicological information, especially to support those findings on hair fibers and this was the main focus of the present work [1]. Thus, this case report describes the practical application of combining hair samples with fly larvae collected from two real cases of skeletonized human remains.

## Case history

The following case report includes two unrelated subjects who were discovered several months after death and were analyzed at the Institute of Legal Medicine of the University of Modena and Reggio Emilia, Italy. The cases are heterogeneous with respect to sex as well as the presence of drugs in the surrounding environment. Details of the two cases are summarized in Table 1.

**Table 1:** Overall information about the two cases covered in this work

Case	Circumstantial data	Decomposition changes
1	Man found under a bridge on the bank of a river covered with cardboard sheets in supine position. Cadaver was entirely dressed. No signs of violence. Presence of a blister of phenobarbital (Xilishupi^®^, PB 10 mg per tablet) in the trouser pocket of the victim.	Advance putrefactive phenomena. Skeletonization of the entire part of the body.
2	Woman found in a wooded area in the city. The body was found in supine position. No signs of violence. No drugs associated with the body or in the surrounding environment.	Advance putrefactive phenomena in the chromatic-colliquative phase. Skeletonization of the upper part of the body.

Both cases described herein were part of a legal investigation in which the identification of the bodies, as well as the cause and manner of death, were requested by a public prosecutor. However, the estimation of the time of death based on entomological evidence was not requested by the prosecutor; therefore micro- and macro-environmental data were not authorized to be collected and not all specimens underwent species identification.

### Case 1

At the beginning of November, the corpse of a man was found under a bridge on the bank of a river near the city of Modena, Italy. The body was found in the supine position and covered with cardboard sheets. The cadaver was fully clothed and exhibited advanced putrefactive phenomena with the head, neck, thorax, legs, and arms skeletonized; therefore, identification of the subject was not possible.

The corpse was completely covered with hundreds of apparently identical species of blowfly larvae, full and empty puparia, and adults. No projectile or fragments thereof were seen on complete body radiographs. Toxicological analyses were performed on fly larvae collected from the thoracic cavity and hair samples collected from the head region. Tables of Xilishupi^®^ (phenobarbital, PB, 10 mg/tablet) were found in the victim’s trouser pocket.

### Case 2

At the end of October, the remains of a woman in the supine position were found in a wooded area also near the city of Modena. The cadaveric human remains exhibited postmortem decomposition with the upper body regions almost completely skeletonized. Identification of the subject was not possible and the remains were completely covered with hundreds of apparently identical fly larvae, full and empty puparia, and adults. There were no obvious signs of external trauma, radiologic investigations revealed no fractures, and X-ray analysis showed the absence of any bullets. Fly larvae were collected from different areas on the corpses, and hair was sampled for toxicological analyses.

## Materials and methods

### Samples for toxicological analyses

All specimens subjected to toxicological analyses were collected by forensic pathologists during the scene investigation (larval samples) and during the autopsy (hair samples). Toxicological investigations were carried out at the forensic toxicology laboratory of the Legal Medicine Institute at the University of Modena and Reggio Emilia. Considering the forensic investigations were requested by a prosecutor for judicial purposes, the approval by an ethics committee was not required.

### Chemical and reagents

Barbital (internal standard, IS), PB, and PHT standard solution (1 mg/mL, methanol) were purchased from Cerilliant Corporation (Merck, Milan, Italy). All stock solutions were stored at -20 °C and used after methanol dilution to appropriate concentrations. All solvents and chemicals for liquid chromatography-tandem mass spectrometry (LC–MS/MS) were of LC–MS purity grade (Baker-VWR, Milan, Italy), while other chemicals used for sample preparation were of analytical grade (Carlo Erba, Milan, Italy). Bond Elut C18 extraction columns (500 mg, 6 mL) supplied from Agilent Technologies (Santa Clara, California, United States) were used for the solid-phase extraction (SPE) procedure.

### Sample Preparation

The case specimens (hair and fly larvae) were subjected to a standard routine systematic toxicological analysis (STA) used in the laboratory. Both hair samples were collected by the coroner during autopsy and a total of 4 cm in length for Case 1 and 10 cm in length for Case 2 were submitted for analysis in the laboratory. Then, the hair samples were then segmented into proximal and distal portions of 2 cm each starting from the scalp end. In both cases, only the first 4 cm were analyzed, and these segments were processed separately. First, both segments were decontaminated as recommended by the Society of Hair Testing guidelines [4]. Briefly, hair samples were washed with acetone (2 × 5.0 mL) and n-hexane (2 × 5.0 mL) and then dried at room temperature. Subsequently, the washed hair segments were pulverized using a Precellys^®^ 24 (Bertin Technologies-Alphatech SpA, Genoa, Italy) at a rotation frequency of 6000 rpm for 30 s. This treatment produced hair fragments of 1–2 mm in length, and the temperature inside the device did not exceed 40 °C, thereby avoiding sample overheating. After this pulverization step, 50 mg aliquot of each segment were weighed into individual vials and subjected to analysis.

The entomological samples colonizing the corpses were collected at the death scene by an authorized agent. The insects were kept in clean and sealed containers to avoid cross-contamination and stored at -20 °C until analysis [11]. First, these samples were abundantly washed with deionized water and dried with filter paper. Then, two aliquots of 50 mg from each case were homogenized using the same Precellys^®^ 24 equipment at the same conditions as those applied to the hair matrix.

Comprehensive screening for licit and illicit drugs (a total of 370 analytes, including metabolites) was performed using LC–MS/MS and full scan gas-chromatography-mass spectrometry (GC–MS). As a result of this STA step, the presented cases screened positive for PB and phenytoin (PHT); therefore, confirmatory analysis was focused on these analytes.

Confirmatory analyses for both sample types consisted of a digestion procedure using a protocol proposed by Frison et al. with modifications. Briefly, the previously weighed 50 mg aliquots of hair or larval homogenate were sonicated at 40 kHz and 30 °C overnight in 2 mL of 0.1 M sodium bicarbonate and 50 µL of IS solution (2 µg/mL barbital). Barbital was selected as the IS after the screening showed no presence of this substance in the samples. Once the digestion was completed, the samples were centrifuged at 5000 rpm for 6 min and the supernatants were transferred to clean tubes where the pH was adjusted to 5.5 by adding 1 M HCl [12]. These tubes were vortex-mixed and subsequently extracted by SPE.

The SPE columns were conditioned with 6 mL of methanol followed by 6 mL of deionized water. Then, the aqueous extracts were slowly loaded onto the cartridges, which were subsequently rinsed with 3 mL of 0.01 M HCl and 3 mL of deionized water. Finally, elution was performed with 3 mL of dichloromethane, and the solvent was evaporated under a gentle N_2_ stream at room temperature. The residues were reconstituted in 100 µL of mobile phase to be injected into the LC–MS system. For quantitation of both sample types, calibration curves and quality controls were constructed by spiking increasing concentrations into 50 mg of blank hair or larval matrices. The appropriate calibration model was determined to be linear with a weighting factor of 1/x and the coefficient of regression was always ≥ 0.998 for both PB and PHT. No sample dilution was necessary. The linear range of this model was 0.2 − 50 ng/mg for PB and 0.3–50 ng/mg for PHT for each matrix. Additionally, detection limits for PB and PHT were 0.1 and 0.2 ng/mg, respectively; quantitation limits for PB and PHT were 0.2 ng/mg and 0.3 ng/mg, respectively, while method accuracy and precision are within accepted range of international validation guidelines (≥ 85%) [13]. These methods are part of a standard, previously validated analytical panel used in the laboratory for the confirmatory analysis of different drugs. Finally, the samples were analyzed in duplicate (*n* = 2) for both hair and fly larvae to assure reproducibility of the concentration found.

### Instrumentation and analytical conditions

LC–MS analyses were performed on an Agilent 1290 Infinity II LC system (Agilent Technologies, Waldbronn, Germany) coupled with a SCIEX API 6500 QTRAP mass analyzer equipped with a Turbo Ion Spray source (SCIEX, Toronto, Canada). LC column was a Force Biphenyl (50 × 3.0 mm, 3.0 μm − Restek^®^, Milan, Italy) preceded by an UltraShield UHPLC precolumn filter (0.2 μm frit) (Restek^®^). Mobile phase A consisted of 0.1% formic acid in water and B 0.1% formic acid in methanol using the following gradient program: 0.0–8.0 min, linear gradient from 4% to 100% (B); 8.0–9.0 min, isocratic at 100% (B), 9.0–9.01 min, linear gradient from 100% to 4% (B). A pre-equilibration period of 1.99 min was used between each run, the flow rate was 0.4 mL/min, and the column temperature was 40 °C. The autosampler was kept at 10 °C and the injection volume was 5 µL. The mass spectrometer operated in negative electrospray ionization (ESI) mode using multiple reaction monitoring (MRM). ESI source settings were: ion-spray voltage: 4500 V, source temperature: 450 °C, nebulization and heating gas (air): 55 psi and 65 psi, respectively. Nitrogen as collision gas was produced by a gas generation system (Nitrogen Generator model 75 − 72, Whatman Inc., Massachusetts, United States). MRM transitions and optimized parameters are presented in Table 2. The Analyst^®^ Software (version 1.7.3, AB SCIEX, Foster City, California, United States) was used for instrument control, data acquisition, and qualitative data analyses. Quantitative data analyses were performed by the SCIEX OS software (version 2.0.1).

**Table 2:** MRM transitions and other instrument-related parameters

Compound	Retention time (min)	Q1 mass (m/z)	Q3 mass (m/z)	DP	CE	CXP	EP
Phenobarbital	5.73	231.0	**85.1**	−80	−17	−5	−10
			188.1	−80	−15	−7	−10
Phenytoin	5.8	251.0	**101.8**	−115	−28	−7	−10
			207.8	−115	−22	−9	−10
			180	−115	−22	−10	−10
Barbital (IS)	4.33	183.0	**139.8**	−60	−16	−5	−10
			85.0	−60	−16	−7	−10

The underlined ions represent the transitions used as quantifier. IS: internal standard, DP: declustering Potential; CE: collision energy; CXP: collision cell exit potential; EP: entrance potential

## Results

In the two particular cases covered by this work, combining the use of toxicological and entomotoxicological findings filled some of the gaps resulted from the absence of traditional biological samples, therefore supporting the medico-legal investigation.

In Case 1, due to the advanced stage of skeletonization, hair was the only available matrix. Additionally, larval samples at different developmental stages were collected and the preliminary toxicological analyses revealed positivity to PB on both sample types. Confirmatory analysis of the hair sample showed the presence of PB on both proximal and distal segments (37 and 24 ng/mg, respectively – Table 3). These findings are in agreement with the circumstantial data found in the subject’s belongings (Xilishupi^®^ tablets), which were later confirmed to contain 6% of PB per tablet. The presence of this compound was also confirmed in larval extracts (25 ng/mg – Table 3).

**Table 3:** Concentration of the drugs in both analyzed samples

	Case 1	Case 2	
Sample	PB (ng/mg)	PHT (ng/mg)	PB (ng/mg)
Proximal hair (2 cm)	37	12.7	19.8
Distal hair (2 cm)	24	N.D.	16.7
Larvae	25	12	35

N.D.: not detected; PB: phenobarbital; PHT: phenytoin

Similarly, in Case 2, the subject was in an advanced putrefactive state, with skeletonization of the upper part of the body. Consequently, hair from the head region and larvae colonizing different areas of the body were collected and submitted for toxicological investigations. PB was found in combination with PHT in both hair and larval samples. However, while confirmatory analysis showed the presence of PB on both proximal and distal segments (19.8 and 16.7 ng/mg, respectively), PHT was only detected in the proximal segment (12.7 ng/mg) (Table 3). Additionally, the larval samples were found positive for both PHT and PB (12 ng/mg and 35 ng/mg, respectively), serving as further evidence of intake of these medications by the subject at least hours before death (Table 3).

## Discussions

Only a limited number of studies reporting the concentrations of PB and PHT in hair or larval samples are available in the literature. Goullè et al. demonstrated that PB concentrations in hair may range from 1.5 to 92.7 ng/mg (with corresponding serum concentrations of 3.5–31.3 mg/L) depending on the posology (10–200 mg/day). Based on this evidence, the PB concentrations detected on the hair fibers of both subjects are consistent with repeated exposure to this drug during the four months preceding death, suggesting regular antiepileptic treatment [14, 15]. The detected PHT concentration, which was found only in Case 2 and in the proximal hair segment, is suggestive of a patient undergoing treatment with this drug, but limited to 1–2 months prior to death, as PHT was not detected in the distal segment (excluding the drug therapy over 2 months before death). In that regard, Tsatsakis et al. demonstrated that the hair PHT concentrations are dose-dependent, and the duration of PHT treatment and gender did not significantly affect these values [14]. Moreover, the hair PHT concentrations declared in this study ranged from 3 ng/mg to 26.7 ng/mg, which correspond to plasma concentrations ranging from 7.2 µg/mL to 19.1 µg/mL. In addition to this study, hair PHT concentrations of 3.3 − 0.1 ng/mg [16], 6.0 to 158 ng/mg [17], and a mean concentration of 13.7 ng/mg [18] have also been reported in other works. Therefore, our findings are in agreement with these previous reports.

Evidence in the literature describing PB concentrations on entomological samples reports somewhat heterogeneous values [19–22]. For example, Beyer et al. [21] published the first report about PB determination in larval samples with a concentration of 100 ng/mg. Kintz et al., on the other hand, found PB concentrations ranging from 0.500 to 0.761 ng/mg [19, 22]. Thus, the concentrations of this drug detected in our samples fall within the range previously described in the literature. Nevertheless, entomological samples should be used only as a qualitative complementary source of information because the amount of a drug in an insect’s organism is highly dependent on factors such as environmental conditions, physicochemical proprieties of the drug, the region of the corpse on which the insect fed, and thanatomicrobiome-related and postmortem phenomena [1, 20, 23, 24]. Hence, it is not possible to establish the dose of a substance to which the subject was exposed solely based on larval findings. As a result, the qualitative entomological evidence obtained during the toxicological investigation of these cases could only be used to indicate a recent exposure of the subjects to PB in the hours preceding death. These findings are ultimately complementary to the hair analysis and supports the conclusion that the subjects had taken the medication relatively close to the time of death.

In both cases, the autopsy performed on the human remains showed the absence of a specific mechanism of fatal injury and the lack of suitable specimens hindered the investigation. As a result, neither autopsy nor toxicological findings were able to establish the cause and manner of death. Nevertheless, the findings obtained from the toxicological investigation for Case 1 matched the profile of an epileptic patient undergoing PB therapy who had been reported missing by authorities four months before his corpse was found. Similarly, the findings obtained from Case 2 were consistent with the profile of an epileptic patient undergoing combined PB and PHT therapy who had disappeared from her home two months before the body was found. The identities of the subjects were then confirmed by comparison of DNA samples extracted from tooth pulp to the saliva of the subjects’ alleged relatives.

Hair is a matrix that remains stable and suitable for toxicological analysis for extended periods, even decades, but it does not provide information on recent exposure [25]. On the other hand, entomotoxicological studies provide knowledge of insects, allowing the correlation of xenobiotics found in these organisms colonizing corpses. Our results do not confirm by any means the cause of death, especially considering that the drug quantities in larvae are highly dependent on factors such as time of sampling and the area of the corpse it was feeding on [2]. Instead, its use solely provided additional information on the drug profile of the subjects, which was in agreement with the results of hair samples (Table 3). Thus, combining these two samples is a valuable tool to identify drugs consumed over both extended and recent periods prior to death, especially in cases where conventional specimens are unavailable, such as carbonized, skeletonized, and/or highly decomposed human remains [1]. In fact, reports show the application of this strategy to various substances, such as benzodiazepines [26], cocaine [20, 27, 28], opiates [20, 22, 26, 29–31], antidepressants [20], and PB [19–22]. However, this approach is still under-researched and requires further documentation and study. Moreover, to the best of our knowledge, this is the first study reporting the presence and concentrations of PHT in entomotoxicological specimens obtained during the toxicological investigation of real forensic cases. Consequently, the lack of data in the literature hinders further interpretation of our findings related to this antiepileptic drug.

## Conclusions

In the present work, hair and larval samples were collected from two independent forensic cases at advanced stages of decomposition. In both cases, these were the only specimens available for toxicological analysis. The successful combination of these biological and entomological samples provided valuable additional information to solve the investigations, as the detection of PB and PHT allowed the profiling of the subjects as patients undergoing treatment with these antiepileptic drugs. These subjects were then traced back to missing persons reports and further confirmed by DNA analysis.

Although a detailed characterization of the insects was not performed in the present work, we nonetheless expect to contribute to the further development of this growing science and its application to forensic toxicology assisting in challenging cases, such as those described herein.
